# Exploring Consumer Preferences and Challenges in Hybrid Meat Products: A Conjoint Analysis of Hotdogs

**DOI:** 10.3390/foods13101460

**Published:** 2024-05-09

**Authors:** Kashmira Salgaonkar, Alissa A. Nolden

**Affiliations:** Department of Food Science, University of Massachusetts Amherst, Amherst, MA 01003, USA

**Keywords:** blended meat, plant-based meat, sustainability, consumer acceptance, food neophobia

## Abstract

Plant-based meat has been the primary strategy to reducing meat consumption. While this category has demonstrated success, with the market value estimated to reach USD 20 billion by 2023, the subsequent reduction in meat consumption has not been proportionate. An alternative approach is hybrid products, which are thought to produce products that more closely resemble meat products. However, whether consumers will be willing to purchase hybrid products remains uncertain. Therefore, the present study uses a conjoint analysis approach to assess the product features driving the selection of a hybrid hotdog. This approach uncovers factors driving consumers’ purchase intentions for hybrid meat products when offered as a choice against 100% plant-based and 100% beef products. In an online survey, participants (n = 454; 45.6% female) were asked to select the product they would be most willing to purchase, varying in four characteristics: protein source, price, fat content, and price. Following this task, participants answered questions related to meat attachment, food neophobia, health, ecological, social, and moral motives regarding food consumption. The results revealed that protein source was the most important factor driving product selection, followed by price, fat, and packaging claims (35%, 24%, 21%, and 20% relative importance, respectively). In this study, hybrid hotdogs were the least preferred to beef and plant-based (−16, −2.5, and 18 part-worth utility, respectively). These product-specific attributes (protein, fat, and price) had distinct relationships with the choices of hybrid, plant-based, and hybrid hotdogs, with these factors together explaining slightly more variability in the selection of hybrid (9%) compared to plant-based (7%) and beef hotdogs (4%). For hybrid hotdogs, protein had the greatest influence (B = −1.2) followed by fat (B = −0.8) and price (B = −0.5). Interestingly, person-related parameters (health, meat attachment, ethics, and food neophobia) had no relationship with the selection of hybrid hotdogs, contrary to plant-based (7%) and beef hotdogs (5%). This influence of the different parameters on the selection of hybrid meat is thought to be due to the lack of consumer knowledge and familiarity with hybrid products. The current understanding of plant-based products may not correspond to hybrid products. Engaging with consumers during the development of these products is critical to ensure consumer acceptance and thus support the transition to a more sustainable diet.

## 1. Introduction

An individual’s dietary patterns and food purchase decisions are influenced by physiological, psychological, physical, cognitive, economic, religious, and sociocultural factors [1]. It is important to gauge the impact of these parameters to determine a consumer’s expectations and requirements while developing products that can be improved versions of existing products or products with new characteristics. This information is also product-specific and varies over time. In the past decade, the alternative meat industry has examined these factors alone and in combination to better understand consumers’ perception and acceptance of these modern plant-based alternatives.

Consumers highly value meat for its nutritional benefits, and it is considered a symbol of heritage, power, masculinity, and pleasure [2,3]. However, within the past decade, there has been an increase in the development of theories that promote a reduction in the consumption of this dietary component [4]. Research in the fields of medicine [5], environmental sustainability [4], and animal welfare [6,7] has encouraged a reduction in meat consumption. However, despite this evidence, many consumers are not motivated or successful in reducing their animal meat consumption [2,8] by switching to plant-based meat alternatives. 

Currently, the primary reasons thought to be driving the lower consumption of sustainable plant-based meat alternative options are centered around the product’s formulation, which impacts the sensory or palatability characteristics, such as taste and mouthfeel [9]. While taste is a primary driver for food choice, meat consumption is also emotionally driven by a phenomenon known as meat attachment [2]. Additionally, since meat has been a popular component of family meals, consumers have also associated several social motives [10] with meat, such as considering it to be the ultimate source of protein [11,12] and, in some cases, also treating it as a necessary component for dinners [13]. 

To overcome these challenges in accepting plant-based meat, several approaches have been considered to improve and more closely align the sensory attributes of meat, either by using processing methods such as extrusion or food printing or modifying the formulation by incorporating various binding agents, flavors, and other components [14]. Additionally, several theories and models have been developed that help manufacturers of plant-based meat products market their products and highlight their sustainability efficiently. These efforts taken by society and food scientists are steadily improving the acceptance of plant-based meat but have not sufficiently caused a reduction in the consumption of animal meat [10].

Another approach to building a bridge between plant-based and animal meat is hybrid meat, which combines plant-based and animal meat components in varying ratios [15,16]. These products provide consumers with alternatives that support a gradual transition from a meat-based to a flexitarian diet. Though some studies suggest consumers have a positive opinion about hybrid products, with a higher preference for these products over 100% plant-based [17,18], there have been reports of negative opinions and skepticism about its formulation and processing by considering them to be artificial, unconventional, and highly processed [16,19]. 

Thus, the purpose of the present study is to assess consumers’ perceptions regarding hybrid meat in further detail and to determine if and how the different product-specific and psychological parameters that have been influential in the consumption of animal and plant-based meat relate to hybrid meat. Understanding the impact of the various parameters through a comparative approach is crucial to determining the major parameters motivating or challenging the choice of a hybrid meat alternative. A comparative understanding of the three meat types based on product-specific attributes will be gauged through conjoint analysis, a market research tool. This tool has been used to measure the influence of different attributes linked to plant-based meat [20,21]. The product selected for the current study is a hotdog, since we wanted to include a familiar meat variety for the cross-protein reference. This is mainly because consumers are better able to determine their preferred meat alternatives when using a popular meat product [22]. The results of this study will help manufacturers of hybrid meat address the gap identified through this study and thus improve the acceptance of hybrid meat.

## 2. Materials and Methods

All procedures were approved by the Institutional Review Board (protocol ID #3647) at the University of Massachusetts Amherst, and informed consent was obtained before testing. This study recruited interested individuals residing in the USA between the ages of 18 and 65. Using Amazon Mechanical Turk (MTurk), we recruited a sample of 571 participants, with 454 meeting the inclusion criteria. Participants received compensation for their time.

### 2.1. Willingness to Purchase

The willingness to purchase was determined using Choice-Based Conjoint Analysis (CBC) [20,23]. CBC is a marketing analysis approach which can be leveraged to uncover consumer preferences using a menu-based approach. Data were collected using Sawtooth Lighthouse (Provo, UT, USA). Participants were presented with product descriptions that provided product profiles for each hotdog based on their protein type, fat content, price, and package claims, as described in Table 1. These attributes were selected based on previous studies involving the conjoint analysis of meat and meat alternatives [20,24,25,26]. Each participant viewed four sets of products, with each set comprising four products. For each set of products, the participants were asked to select the product they were most likely to purchase.

### 2.2. Psychological Questionnaires

The psychological questionnaire comprised 51 statements. The factors evaluated in this questionnaire were meat attachment, food neophobia, and the respondent’s agreement with food-specific health, ecological, moral, and social motives. All these factors were measured on a 5-point Likert scale (i.e., 1 = totally disagree, 2 = disagree, 3 = neither disagree nor agree, 4 = agree, 5 = totally agree).

#### 2.2.1. Meat Attachment

The meat attachment questionnaire developed by Graca and colleagues (2015) [2] was employed in this study and comprised 20 statements. This questionnaire evaluated an individual’s attachment to meat through a four-factorial construct, which included measures such as Hedonism (referring to a high meat consumption for the purpose of pleasure), Affinity (attaching a high degree of affinity to meat consumption), Entitlement (representing the feeling of having a right to consume meat), and Dependence (referring to the feeling of dependence on meat consumption).

#### 2.2.2. Food Neophobia

The food neophobia scale developed by Pliner and Hobden (1992) was used to assess consumer attitudes towards novel food products and their consumption [27]. The question block comprised 10 statements to evaluate the phenomenon.

#### 2.2.3. Health, Ecological, and Moral and Social Motives

The health (7 statements), ecological (6 statements), moral (5 statements), and social (5 statements) motivations for consuming plant-based meat were determined through statements included in the study conducted by Pires and colleagues (2019) [28].

### 2.3. Demographics

Participants also answered questions regarding their age, gender, level of education, employment status, diet, frequency of animal-based and plant-based meat consumption, and number of children and family members. These variables were selected based on previous research examining meat attachment and consumer attitudes towards plant-based food [2,26].

### 2.4. Data Analysis

The relative importance of product factors such as protein type, package claims, price, and fat content for selecting different hotdogs was determined using the Hierarchical Bayesian Model [29] through the Sawtooth Lighthouse software package (https://sawtoothsoftware.com/lighthouse-studio, Provo, UT, USA). The preference for each level under the different labels was expressed in terms of zero-centered part-worth utilities [30,31]. For person-related values, the average scores were calculated for meat attachment, food neophobia, health, social, moral, and green values, following the recommendation of prior literature examining these parameters [32]. A multiple linear regression was conducted to determine the impact of different product-specific and psychological parameters on the preference level for the hybrid hotdogs and to compare the impact against 100% Beef and 100% Plant-based. For this analysis, the moral, social, and green values were combined and labeled as ethical values. This analytical approach follows prior studies [6,33,34]. R studio (version 4.1.2) was used to conduct data analysis for the survey.

## 3. Results

Of the 454 participants who met the inclusion criteria in our study, 54.1% were male, and 45.6% were female. Roughly half of the participants (48.4%) were between the ages of 30 and 50, with 39.9% in the age group of 18–29, while the remainder (11.7%) were in the age bracket of 50–65 years old. The majority of the participants (95.2%) reported being primary grocery shoppers for their household and had at least one child (77.3%) in their family. Regarding education, most participants (87.9%) at least had a bachelor’s degree, and 95.8% reported being employed. Participants were familiar with both beef and plant-based hotdogs, with 56.8% reporting consuming plant-based hotdogs and 81.1% being consumers of beef hotdogs at least once weekly. Participants were asked to select which product (plant-based, beef, or both) was healthier, and the results showed that a similar number of participants considered plant-based meat and conventional meat to be healthy (34.5% and 31.9%, respectively). In terms of environmental friendliness, plant-based meat was chosen by 64.3% of the participants over meat, while 28.2% considered meat to be more environmentally friendly than plant-based meat.

### 3.1. Comprehensive Product Selection

**Figure 1:**
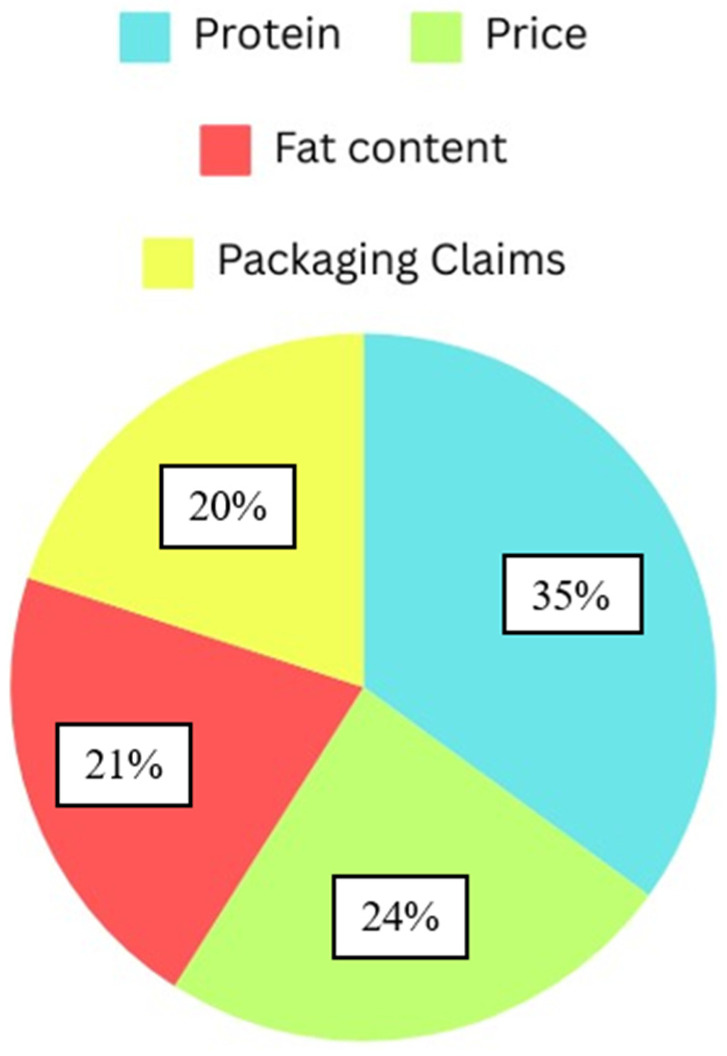
Relative importance of product-specific attributes.

**Figure 2:**
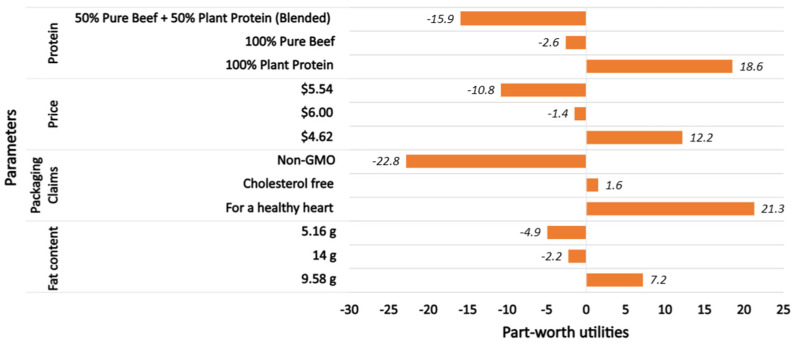
Part-worth utilities for product-specific attributes explaining the preference for different levels under each attribute.

The Hierarchical Bayesian Model determined the zero-centered part-worth utility for each level of the different attributes and the relative importance of every attribute (Figure 1 and Figure 2). Regarding relative importance (Figure 1), protein source (35%) was found to be the most important factor influencing the product selection. Hotdogs containing 100% plant protein were preferred the most, and the hybrid variety was found to have the lowest utility value. Price (24.5%) was the second-most influential factor in product selection, while Fat content and Package claims (20.5% and 19.4%, respectively) were found to influence the product choice equally. Based on the utilities presented in Figure 1, it was found that participants had a higher preference for hotdogs with medium to high fat levels, while hotdogs carrying the claim ‘For a healthy heart’ had a higher preference than those that stated to be ‘Non-GMO’ and ‘Cholesterol free’.

### 3.2. Product-Specific Parameters

**Figure 3:**
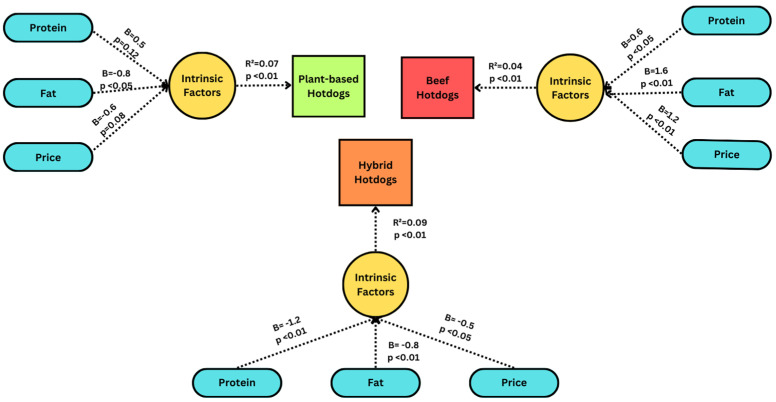
Influence of product-specific parameters on hotdog varieties. Three separate regression models were performed, one for each product type (plant-based, beef, and hybrid). R^2^ and respective *p*-values are reported for the entire model, while beta (B) (and *p*-values) are reported as individual variables.

A multiple linear regression analysis was performed to determine the relationship between product attributes and hotdog selection (Figure 3). The dependent variables included under the intrinsic model were the values of relative importance for protein, fat, and price. The model for hybrid hotdogs explained 9.3% of the variance via all three intrinsic product attributes (β_protein_ = −1.2, β_price_ = −0.5, β_fat_ = −0.8) as significant negatively influential parameters. For plant-based hotdogs, one significant parameter was fat, which had a negative effect (β_fat_ = −0.8), and the model explained 6.6% of the variance. Protein (*p* = 0.12) and price (*p* = 0.08) had no significant effect on the selection of plant-based hotdogs. The model for beef hotdogs included protein (β_protein_ = 0.6), fat (β_fat_ = 1.6), and price (β_price_ = 1.2) as significant predictors, and the model explained 4.3% of the variance. 

### 3.3. Psychological Parameters

**Figure 4:**
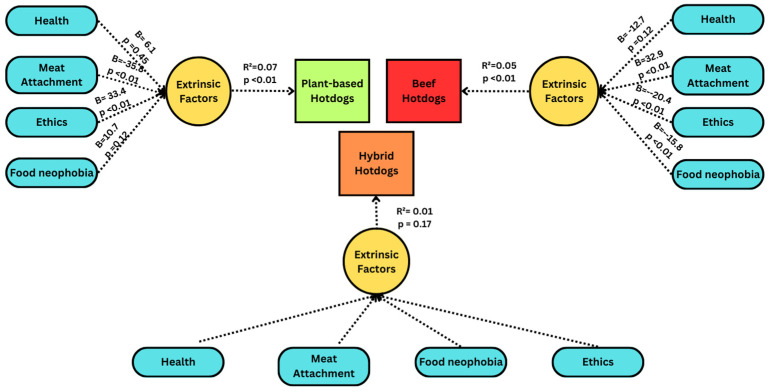
Influence of psychological parameters on hotdog varieties. Three separate regression models were performed, one for each product type (plant-based, beef, and hybrid). R^2^ and respective *p*-values are reported for the entire model, while beta (B) (and *p*-values) are reported as individual variables.

A similar multiple linear regression analysis was performed to determine the relationship between extrinsic attributes and hotdog selection (Figure 4). The dependent variables included under the extrinsic model were scores for food neophobia, meat attachment, and health and ethical motives. The model for hybrid hotdogs was not significant (*p* > 0.05). For plant-based hotdogs, meat attachment (β_meat attachment_ = −35.8) and ethical motives (β_ethical motives_ = 33.4) were the significant parameters and explained 6.6% of the variance in the selection of a plant-based hotdog. For beef hotdogs, 5.2% of the variance was explained by the model with meat attachment (β_meat attachment_ = 33.0) and food neophobia (β_food neophobia_ = −15.8) as the significant parameters.

## 4. Discussion

Consumer preferences for hybrid hotdogs compared to plant-based and beef hotdogs were surveyed to investigate the influence of product-specific and psychological parameters on product choice. The understanding of how these parameters drive the selection of plant-based meat, hybrid meat, and beef was explored to better understand the potential of hybrid meat products to be accepted by consumers. In terms of food habits, participants in the present study reported consuming plant-based meat as frequently as beef. This result was supported by the conjoint analysis results, wherein plant-based hotdogs were most preferred, followed by beef. However, the lowest preference for hybrid hotdogs was contrary to past studies where consumers considered hybrid meat as the first step in meat reduction and thus recorded higher acceptability scores and purchase intent for hybrid meat over plant-based meat [18,35]. This contradiction in the preferences may be driven by the demographics of our participants, who reported a higher preference and consumption frequency for plant-based meat. In this study, participants were not recruited based on dietary habits, outside of consuming hotdogs.

To better understand this low preference of hybrid hotdogs, we examined the relationship between product-specific parameters and product selection. Here, we observed that the choice for hybrid hotdogs was associated with protein source and fat, while for plant-based hotdogs, though protein source was not a significant attribute influencing its selection, fat had a significant negative effect. However, in terms of beef hotdogs, both protein source and fat content had a significant positive impact. The missing significance of protein source in the case of plant-based hotdogs can be tied to the incomplete amino acid profile of plant proteins against the complete 20 amino acids in meat [36]. Dieticians describe this difference as not providing the desired protein content [35]. Regarding the fat content, the participants from our survey had the highest preference for hotdogs containing 9.58 g, which was the medium fat level considered for the study. Since plant proteins generally have lower fat content, the negative association can be explained [36].

Despite the difference in protein source and fat content influencing the selection of beef and plant-based meat, there was a similar percentage of participants who considered plant-based meat to be healthier than meat and vice versa. This is comparable to previous results, where one study identified that 33.6% of respondents considered both plant-based and animal meat to be equally healthy [37]. As a result, neither for plant-based nor for beef hotdogs were health motives found to be an influencing factor affecting the choice. This absence of health motives as an influencing parameter can also be attributed to meal context which, in our study, was hotdogs, often regarded as a non-healthy food choice, and several studies have proven that meal context influences the motivations supporting food consumption [22,38].

The second most important intrinsic attribute in the conjoint analysis after protein was price, which was found to restrict the selection of hybrid hotdogs and had a significant positive effect on the selection of beef hotdogs but was not significant in the selection of plant-based hotdogs. The variation in price as a parameter affecting choice may be attributed to various factors such as the current market share of the product as well as consumers’ consumption frequency. While plant-based meat is widely available in grocery stores, hybrid meat still has limited market visibility. Additionally, hybrid meat also showed a negative correlation with other product-specific attributes, which highlights that consumers might not be willing to pay for the product. On the contrary, our participants were also frequent-to-daily consumers of plant-based meat; therefore, the price barrier may not be a grave concern, as seen in past studies [39,40]. Thus, participants in our study tended to be influenced by price only in the case of beef and hybrid meat but not plant-based meat.

Identifying the psychological factors that have been crucial in affecting the acceptance of plant-based meat, correlating them to hybrid meat, and comparing the correlation to beef and plant-based meat will help to determine how consumers perceive hybrid meat as a competitive category to these two existing varieties. However, the model of psychological parameters for hybrid hotdogs was not significant in our study. In contrast, the models for plant-based and beef hotdogs were significant, with meat attachment and ethical motives as the significant parameters. 

Meat attachment, or unwillingness to reduce meat consumption, has been reported to be a major barrier to substituting animal with plant-based meat [37], which agrees with the present findings. Plant-based hotdogs had a significant negative association with meat attachment, while the selection of beef hotdogs was driven by a significant positive correlation. Interestingly, meat attachment was not a significant predictor of the selection of hybrid hotdogs, suggesting that this product may provide an acceptable alternative for individuals with high meat attachment. Nonetheless, previous work suggests that consumers are skeptical of hybrid meat products that closely replicate plant-based meat or resemble animal meat [16,19,41,42]. This consumer confusion thus translates in the missing effect of meat attachment in hybrid meat, since meat attachment includes all the emotional factors that consumers associate with meat [19]. 

Ethical norms, comprising social, moral, and environmental values, have appeared to be a barrier to the acceptance of plant-based and hybrid alternatives [43,44,45,46]. This reasoning is strengthened by consumer studies, which found that hybrid meat was positively perceived under blind testing; however, it had the lowest preference during informed testing conditions [46,47]. It is suggested that low awareness, familiarity, and an uncertainty of this product category have a negative influence on consumers’ perception regarding the social or moral impact when considering hybrid products. Social perception has been a significant factor in product selection, as consumers do not wish to be marginalized for their food consumption choices [48,49,50]. This reasoning has been derived from studies conducted for plant-based alternatives, which showed that consumers were willing to consume plant-based alternatives at home but not on social occasions [9,49]. In the present study, ethical norms were not significantly associated with selecting hybrid hotdogs. Conversely, our findings demonstrate that the selection of plant-based hotdogs was ethically motivated, whereas a negative relationship was reported for beef hotdogs. This positive association has been replicated in several studies that argued that for an increased awareness of the altruistic benefits of plant-based alternatives; thus, the scenario has been reversed, with evidence demonstrating that consumption may be socially motivated [7,51,52,53].

In the present study, 64.4% of participants considered plant-based meat more sustainable than animal meat. Thus, it was expected that environmental values would have a significant positive relationship with the selection of plant-based hotdogs. However, this effect was not observed in the present study. Additionally, there was no association observed for the selection of hybrid hotdogs. This lack of association is consistent across the literature, which has been linked with consumers’ lack of awareness of the environmental impact of food. Additionally, studies have demonstrated that sustainability is the least important when making a food selection [54]. While the perceived degree of processing was not a focus of this study, consumers’ perception of processing has been described as being associated with increased ecological burden and may not align with perceptions of sustainability [55]. Thus, individual’s food choices is likely driven by a combination of features of the product and person-related values, yet the relationships between these factors are specific to the food product itself. Future studies are needed to uncover these relationships in the context of other plant-based and hybrid alternatives. 

The present consumer survey uncovered a new understanding of consumers’ preferences for hybrid hotdogs compared to plant-based and beef options. Here, hybrid hotdogs were described as a 50–50 combination of plant protein and beef. The ratio blends of actual products are anticipated to vary, with ideal ratios selected through sensory properties [56]. Further, prior work demonstrates that labeling and naming products can influence the acceptance of these hybrid products, which was not assessed in the present study. Future studies are needed to understand how the naming and description of products influence the expectations and liking of hybrid products. The approach considered the importance of meal context on consumer preferences and attitudes. Yet, it is anticipated that the acceptance of hybrid products will differ across product categories, such as burgers or milk. The conjoint analysis approach provides important new insights into the promise of hybrid products for future product development, with opportunities to utilize sensory and consumer studies to understand the complex framework driving the acceptance of sustainable food products.

While hybrid products are considered advantageous from a formulation perspective, addressing limitations in the nutrient profile and sensory characteristics, the present study supports previous reports that consumers report a lower preference for hybrid meat than meat and plant-based options. Product-specific attributes, specifically protein source and fat content, and person-related values, specifically ethical norms and meat attachment, are significant drivers of plant-based and animal hotdog choices, which were not observed for hybrid products. Thus, this can be considered a bold indication of a lack of knowledge and familiarity with the product, and it needs to be addressed effectively. 

## 5. Conclusions

The present study aimed to identify factors influencing consumers’ preferences for hybrid hotdogs compared to plant-based and beef options by including product-specific and psychological parameters. It was found that product-specific parameters were important for the selection of hotdogs, and they were more important for the selection of hybrid compared to beef and plant-based options. Alternatively, psychological parameters were not associated with the selection of hybrid hotdogs, but they were associated with beef and plant-based hotdogs. The most influential psychological parameters were ethical norms and meat attachment, which had contradictory influences on plant-based and beef hotdogs. The missing impact of these parameters can be associated with the lack of knowledge and familiarity about hybrid meat; therefore, consumers relied on product-specific attributes. The findings in this study provide significant insights into consumers’ perceptions of hybrid products relative to animal- and plant-based alternatives. A comprehensive understanding of how consumers perceive hybrid products serves as a foundation for informing future research and facilitating the development of effective strategies to promote the consumer acceptance of hybrid products. This work will aid in advancing and adopting a more sustainable diet.

## Figures and Tables

**Table 1:** Parameter and levels for hotdog profiles.

Parameters	Levels
Protein Type	100% Beef
	100% Plant-based
	50% Beef + 50% Plant-based
Price per 12 oz (8 Franks)	$4.62 $5.54
	$6.00
Fat Content per Frank (43 g)	5.12 g 9.58 g
	14 g
Package Claims	Cholesterol-free Non-GMO
	For a healthy heart

## Data Availability

The original contributions presented in the study are included in the article, further inquiries can be directed to the corresponding author.
